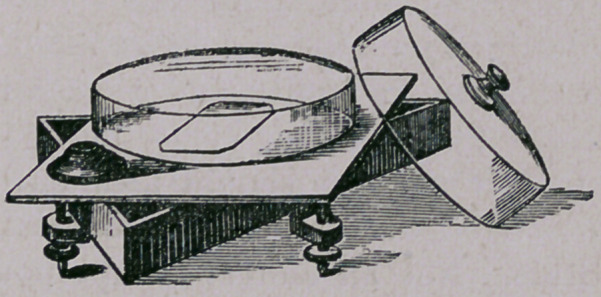# Bacteria Culture

**Published:** 1885-07

**Authors:** F. S. Billings


### Art. XVIII.—BACTERIA CULTURE.

BY DR. F. S. BILLINGS.

While this subject was introduced by Prof. Johne in the last number of the Journal, it is still of so much importance, and at present so much the rage in medicine, that a further description of its methods seems called for. In all bacterial work the first thing which falls upon us to do is Sterilization. If "Cleanliness is next to Godliness" in society, it is even more than that—it is, above all and everything, the first principle and everlasting rule of bacterial culture and studies.

To Sterilization the first thing necessary is the cleanliest washing possible of all glass, earthen, or tin vessels. After being thus washed, they must be rinsed out with a corrosive sublimate solution (1 : 1000 aq.), then with alcohol, and lastly with distilled water. Flasks, test tubes, and such glasses as will bear heat, should be additionally sterilized in this way also. Iron or steel implements can be sterilized by dry heat only. For the ordinary purposes of the practical doctor, the house oven can be made to answer, if it is carefully watched. The flasks and test tubes, after being washed and rinsed as above, are first to be placed, mouth down, in some iron or glass vessel, and dried in the oven, then they must all be corked with cotton plugs (absorbent), which should not fit too tightly; they are then to be placed in the oven and subjected to the action of heat until the cotton is slightly browned; the processes should occupy an hour or even longer, so that all germs that may have been in the air within the vessels have been killed; they can now be allowed to cool, and laid aside, and will remain sterile so long as the cotton plug is not removed and the outside air allowed to enter them *en masse.* For those who intend to make detailed studies of bacterial life, or who are engaged in teaching, it is better to have a proper set of sterilizing ovens and implements.

The accompanying cut represents such an oven, which is double-walled and can be made of heavy tin, sheet-iron, or copper. There should be about one inch and a half space between the two walls. The door must be the same as the sides, top and bottom. In the inside should be ledges for two sliding shelves punctured for test tubes, as seen in illustration. The outside bottom should be of heavy copper, in order to withstand the heat. The oven must be so made that this can be replaced when burnt out. The oven here shown has two openings for thermometers, and a damper or regulator on the top. It is made to hang up on hooks, but must be hung away from wood or combustible material.

This oven is 45 cms. high, 28 broad and 28 wide; it requires three burners to heat it up to 150 or 160° Celsus, and costs in Berlin $12.00, when made of heavy sheet iron.

In order to make use of steam as a means for sterilizing many things that we cannot subject to a dry heat without injuring them, we have a cylindrical vessel, made of heavy tin or copper, covered with a heavy felt or asbestos jacket, and about 3 feet in height and 10 inches inside diameter. The accompanying cut represents such an apparatus.

This is useful in sterilizing everything made from gelatine or agar-agar, potatoes or other vegetables, meats, etc., but is not suitable for blood serum. It is supplied with a thermometer. The water is poured in at the top, and not between the walls, and about 1-3 from the bottom is a grate-work on which to set the vessels. This instrument is also convenient in the making of all gelatines, for we can pour them into one large funnel, place it in a flask and in the steam apparatus, fill the latter, place the Brunsen under it and go home and find our gelatine all ready in the morning to pour at once into the test tubes, which should be filled to about 1-3.

For the manner of preparing gelatines the reader is referred to the last number of the JOURNAL, pp. 120 and 121. In preparing potatoes or vegetables, the eyes or any indentations or hoe cuts, and such like, must be first cut out with a clean knife, sterilized in the Brunsen flame, the cut going into the sound substance, and after that scrubbed with a nail brush in corrosive sublimate.

Another cultivating medium is blood serum, which is the most difficult of all to prepare, and requires great delicacy in treatment, so as not to cause the albuminum to coagulate, thus giving us an opaque instead of an amber-colored gelatinous mass.

Every student of medicine should know how to obtain blood serum, and how the placenta sanguiness is formed and the serum separated. Sheep's blood is the best and horse's the poorest for our purpose. Take several tall glass vessels, with ground glass stoppers, sterilize as directed,—vessels should be at least 15 inches high and 6 in diameter,—proceed to slaughring house; if possible have a servant there to wash the necks of two or three sheep first in water and then in sublimate solution, and again in water; have the head well held back; a clean, quick cut, and catch the blood only so long as it runs strong and quickly—close the vessel and wash it off at leisure. The vessels should then be placed in an ice chest for two or three days, until the serum has become thoroughly separated. It is then to be as carefully drawn off as possible with sterilized pipettes, the cover of the glass not being moved more than necessary—sterilized test tubes are to be filled about 1-3.

In filling all test-tubes or flasks the operator must be extremely careful not to touch the sides of the opening of these vessels with the material he is pouring in, for the cotton plugs will then adhere and be troublesome on removing them for inoculation of the tubes. White, not grey, filtering paper must invariably be used. After having filled our tubes with the serum, we come to the difficult parts of the operation, which are:

1. To sterilize the serum without causing it to solidify.
2. To cause it to solidify without coagulation of its albumen occurring.

For this purpose we have the apparatus designed by Koch, and illustrated by the following cut:

This is a double-walled apparatus, made of copper or block tin, covered with a jacket of felt or asbestos. The cover is also constructed in the same manner. It is divided internally into four departments, for the better arrangement of the test tubes, which are placed standing inside. The walls and top are filled with water, the cock and glass at the left being to draw off and indicate the amount of water. The stand at the right, with the Bunsen, is to heat the water in the cover. The thermostat itself is heated from the bottom. In the cover are places for the thermometer. The test tubes, containing blood serum, are placed in this apparatus for about two hours daily for five consecutive days. The temperature must not be raised during this time to over 60° C. If at the end of that time we desire to use the gelatine as it stands in perpendicular tubes, we simply raise the heat very slowly to *not over* 75° C. or under 70°; the gelatine will then become solid; to be tested by shaking a tube a little.

We most frequently, however, desire to use both agar-agar and serum in tubes, with the gelatine offering all the surface possible to our use; to this purpose we must have an apparatus that will allow us to sterilize them both in an inclined position, but not such that the material in the tube will run up against the cotton plug.

The apparatus must also be a thermostat, for with the serum we have not much freedom in the temperature we can use.

For this purpose we have the following apparatus made of block tin, with glass cover, double walled, covered with felt, and legs capable of raising or lowering on one end. It is supplied with thermometers, water gauges and stop-cocks, and is heated from underneath. A test-tube, filled with water or any fluid, should be used to test the incline we require for our tubes. In sterilizing the agar-agar little care is necessary about the heat. An hour a day for four or five days, and then leave the tubes to cool in the apparatus, which will hold one hundred 5-inch tubes, is all that is necessary.

With blood serum, however, all the previously mentioned precautions are necessary, and the same temperature must be adhered to.

As very many pathogenetic or questionable bacteria do not develop in ordinary room temperature, but in that of the animal body, it has been found necessary to make an apparatus by which we can for months or years keep up a constant degree of temperature at all times without it being subject to variations.

Such an apparatus is called a thermostat, and is made of block tin, double walled, supplied with gas and water gauges, thermometer, etc. It is covered with heavy German felt

This cut represents a thermostat 40 in. long, 15 in. high, and about 20 in. wide. The gas apparatus at the side is to heat it. It consumes but little.

There is one objection to these square thermostats as well as all round ones opening from the top. When necessary to open them the entire chamber is at once cooled off. This can be obviated in the round one if made of copper, by having it quite large and high, and internally divided with double water-holding partitions into three or four compartments each with its own door, each compartment should have its own thermometer, and I am convinced that we can get made such thermostats either round or square, made in compartments, each of which can be held at any desired temperature without influencing the others. Such an apparatus is necessary, and would do away with the necessity of having to have a different thermostat for every variety of warmth one desires; especially is this necessary when we desire to test the action of various grades of heat on the life of germs.

An ice chest is indispensable in such a laboratory.

The above cut represents a triangular oak frame, each arm being 12-in. long. It is arranged with screws, so that it can be made perfectly level. Upon this frame is a square piece of plate glass and underneath should be a dish holding chopped ice, so that the gelatine shall cool quickly. In the dish is to be seen the square of glass, upon which we pour the gelatine (it must never be allowed to run to the edge of plate)—see page 122, April number. The cover should lap over, not set inside, as some of these vessels have been made. By the use of glass bridges ‖————‖ made thus, and a little shorter than the glass plates. We can place five or six plate cultivators in one such vessel.

I have thus described every essential to bacteria cultivating, except the straining methods. For the latter I refer our readers to the manual by Friedlander, "Microscopic Technique," (D. Appleton & Co.) With what has been here detailed and Prof. Johne's paper, and the editorial of the last number in connection with Prof. Friedlander's book, any one who desires can, with time, patience and the necessary means, become an adept in bacteria culture.

## Figures and Tables

**Figure f1:**
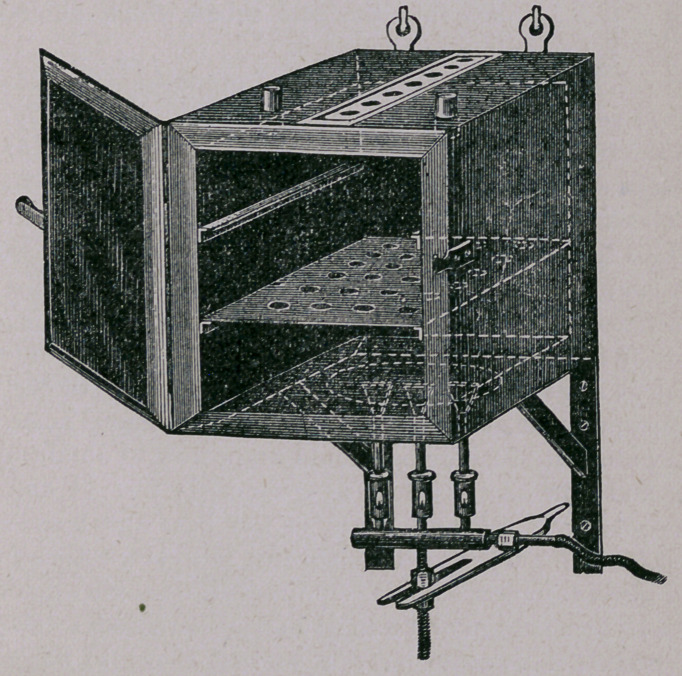


**Figure f2:**
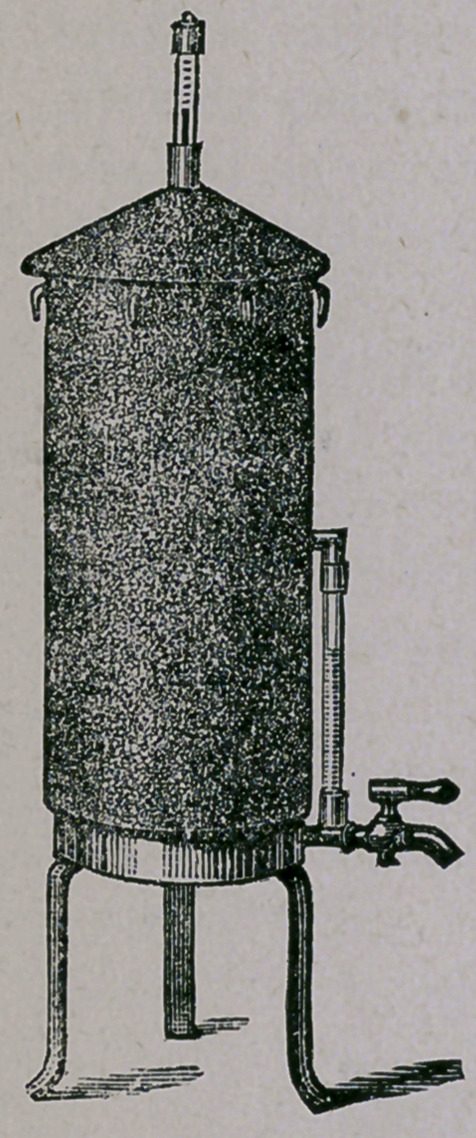


**Figure f3:**
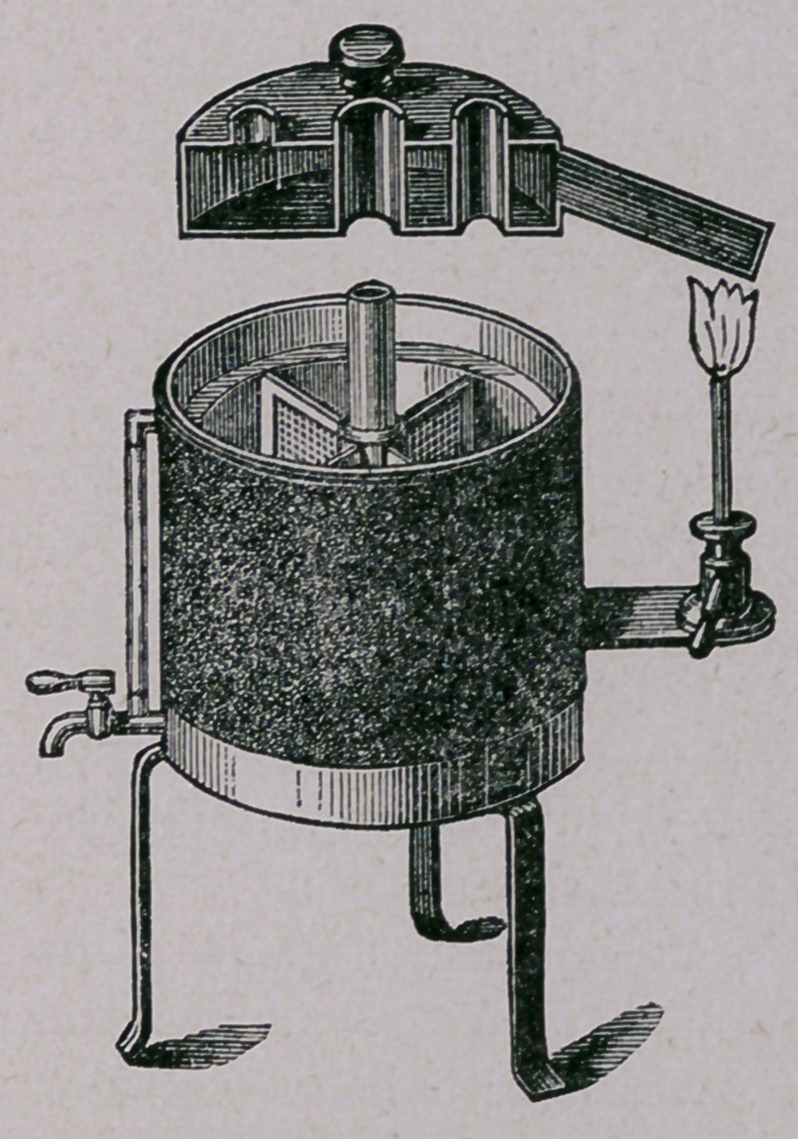


**Figure f4:**
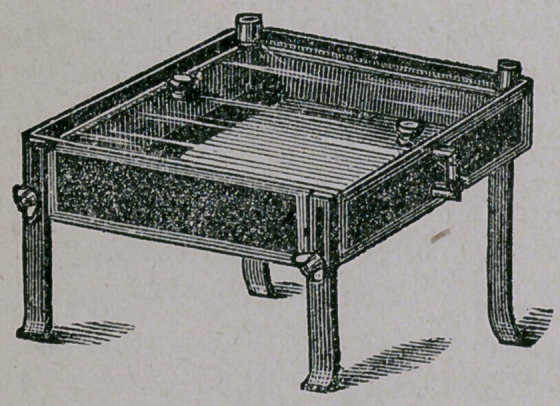


**Figure f5:**
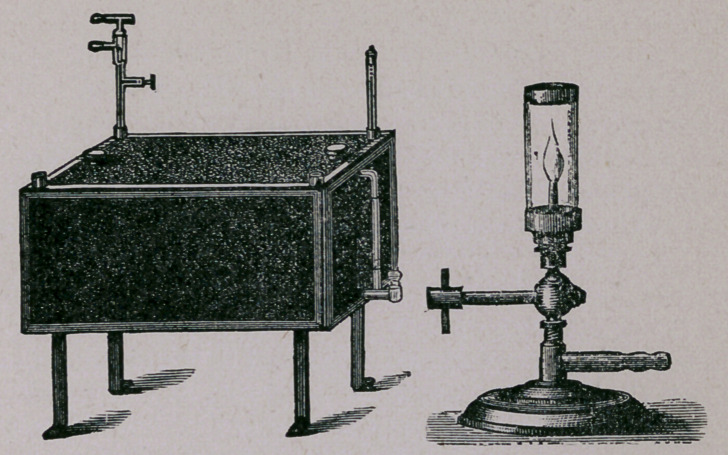


**Figure f6:**